# Polymerase Chain Reaction-Assisted Evaluation of the Efficacy of Seed-Treatment Prevention of *Sporisorium reilianum* Infection in Sorghum Seedlings

**DOI:** 10.3389/fmicb.2021.745144

**Published:** 2021-10-29

**Authors:** Zhi Zhang, Juan Fan, Mucai Feng, Hongbo Qiu, Anlong Hu

**Affiliations:** ^1^College of Agriculture, Maize Institute, Guizhou University, Guiyang, China; ^2^College of Agriculture, Crop Protection Institute, Guizhou University, Guiyang, China; ^3^Agricultural Technology Extension Center, Weifang, China

**Keywords:** sorghum, head smut, seed treatment, PCR, fungicide

## Abstract

Head smut, caused by *Sporisorium reilianum* [(Kuhn) Langdon and Fullerton], is a major disease of sorghum. Seed treatment is considered to be the most effective way to control the disease; however, the pathogen can infect at the seedling stage and the infected plant will not display symptoms until the reproductive stage is reached. The evaluation of the efficacy of seed treatments is time consuming and is dependent upon visible symptoms. Polymerase chain reaction (PCR) methods have the ability to identify pathogens and diagnose their presence at an early stage of infection. In this study, the *S. reilianum-*specific primer SR3 was used for PCR detection pathogen. We optimized temperature, humidity, and spore quantity test conditions and were able to achieve >88% infection incidence in sorghum seedlings. Sorghum seeds were soaked in various concentrations of tebuconazole and planted for 7 days in soil containing 0.2% teliospores. The efficacy of tebuconazole against *S. reilianum* was evaluated by PCR and recorded as disease incidence. Results indicated that the reduction in disease incidence after exposure to 0.15, 0.30, 0.45, 0.60, and 0.75 μg/mL tebuconazole was 6.24, 37.48, 67.74, 81.24, and 93.74%, respectively. Significant differences between the concentrations of tebuconazole were observed. The PCR assay represents a valuable tool for evaluating the efficacy of fungicide seed treatments for the control of *S. reilianum* in sorghum under laboratory conditions.

## Introduction

Sorghum [*Sorghum bicolor* (L.) Moench] is the fifth most important cereal crop produced globally (Mengistu et al., 2019). It is a source of animal feed and fodder, used in traditional and processed foods and beverages, and in the production of biofuel. Head smut of sorghum, caused by *Sporisorium reilianum* (Kuhn) Langdon and Fullerton [syn. *Sphacelotheca reiliana* (Kühn) G.P. Clinton and Sorosporium reilianum (Kühn) McAlpine], is an economically important disease of sorghum worldwide (Zhang et al., 2011). The pathogen can infect both maize and sorghum. Although the fungus is a biotroph, infection of a plant results in a complete loss of any harvestable seed (Poloni and Schirawski, 2016). Teliospores overwinter in the soil and crop debris and provide primary inoculum for the next cycle of infection. Teliospores are released can remain viable for at least 3 years. Teliospores can also adhere to seed surfaces and during seed storage remain viable for much longer periods. The presence of the pathogen is very difficult to eradicate from the soil once it has become established in a cultivated field (Little et al., 2011).

Current disease management strategies include the use of host resistance and integrated pest management (IPM). Integrated pest management strategies that emphasize reduced pathogen pressure, providing optimal plant growth conditions, and the utilization of disease resistance (or tolerance) represent the future trend in disease control (Anitha et al., 2020). The use of crop protection products is the most common approach used to control *S. reilianum* as most commercially available hybrids lack a high level of resistance. In this regard, seed treatments can be used to reduce the subsequent infection of young seedlings and represent an important component of IPM systems. A variety of seed treatments have been developed to reduce pathogen and insect damage, promote uniform stand development, and increase seedling vigor. Fludioxonil, tebuconazole, sedaxane, triadimenol, and azoxystrobin are chemical fungicides that are commonly used as seed treatments (Lamichhane, 2020).

*Sporisorium reilianum* infects sorghum at the seedling stage, when they are only 1.5–2 cm long (Matheussen et al., 1991). After infection, the pathogen continues to proliferate in sorghum tissues without inducing any symptoms in vegetative tissues. The disease manifests itself when the host plant becomes reproductive, and symptoms then become readily visible. In the majority of cases, however, control measures at this stage are not effective. Therefore, developing a convenient, rapid, and accurate detection method for sorghum head smut infection is crucial. Chlorotic flecking along the midrib of maize leaves has been reported as a primary symptom of *S. reilianum* infection (Matyac and Kommedahl, 1985a). *Sporisorium reilianum* hyphae has also been observed in the epidermal tissues of maize coleoptiles stained with cotton blue (Shen et al., 2006). These assessment approaches, however, have not proven to be consistent.

Polymerase chain reaction (PCR)-based methods can be very effective in confirming the presence of a pathogen and detect infections before symptoms appear (Martinelli et al., 2015). In this regard, PCR-based assays for the detection development of head smut of maize caused by *S. reilianum* are readily available, sensitive, and reliable (Xu et al., 1999; Shen et al., 2006; Ren et al., 2008; Fessehaie and Munkvold, 2010).

As noted, the use of seed treatments with fungicides are a primary method for controlling *S. reilianum*. Since the disease does not show symptoms until the reproductive stage of plant growth, however, conventional methods of evaluating the efficacy of seed treatments using field trials is time-consuming and labor-intensive. Therefore, to improve the ability to evaluate the efficacy of fungicidal seed treatments, Anderson et al. developed a real-time PCR-based seedling assay for *S. reilianum* in maize. In that study, they compared five commercially available formulations of fungicides used as seed treatments for their ability to reduce seedling disease incidence using a PCR analysis of root and mesocotyl tissues (Anderson et al., 2015).

The studies of Anderson et al. (2015) focused on the detection of *S. reilianum* in maize at the seedling stage using PCR technology to determine the efficacy of seed treatments. The extension of their approach to the detection of *S. reilianum* in sorghum, however, has not been investigated as of now. In fact, due to the slow growth of mycelia on culture media and the low germination rate of teliospores (Shetty and Safeeulla, 1979; Qian, 1990), few lab studies have been conducted on the efficacy of fungicides against *S. reilianum*. Currently, tebuconazole, applied as a seed treatment, is a major strategy used to protect sorghum seedlings from *S. reilianum* (Yang et al., 2014). In the present study, a PCR assay was used to detect the presence of *S. reilianum* in sorghum seedlings and determine the efficacy of a sorghum seed treatment with tebuconazole in preventing the establishment of *S. reilianum* in sorghum seedlings.

## Materials and Methods

### Sorghum Seeds and *Sporisorium reilianum* Culture

Seeds of the glutinous sorghum variety Qiangao 8 were obtained from the Guizhou Subcenter of Chinese Wheat Improvement. The seeds were washed with tap water to remove any fungicidal residues from the surface of the seeds and then dried at room temperature. The dried seeds were then surface sterilized with 1% sodium hypochlorite in 10% ethanol for 10 min, rinsed with sterile distilled water three times, and immersed in sterile distilled water for 4 h. This was followed by a hot water treatment in sterile distilled water at 60°C for 5 min to kill any endophytic fungi inside the seeds. Lastly, the seeds were rinsed with sterile distilled water, air-dried, and stored at 4°C until use (Huang and Backhouse, 2005).

**FIGURE 1:**
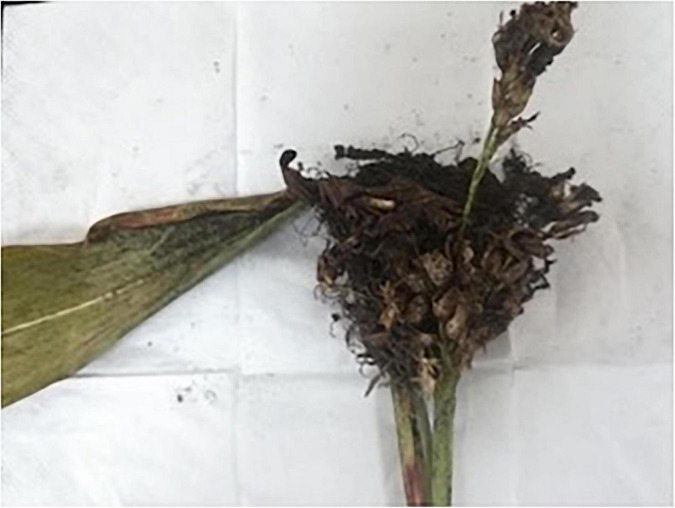
Characteristic symptoms of sorghum head smut caused by *S. reilianum* in naturally infected, smutted panicles of sorghum plants growing in Qianxi county, Guizhou Province, China.

Teliospores of *S. reilianum* were collected in August 2020 from naturally infected, smutted panicles of sorghum plants growing in Qianxi county, Bijie City, Guizhou Province, China (Figure 1). Smut galls were broken up and sieved through a 40-mesh sieve to remove plant material and other debris. Teliospores were collected and then air-dried in paper sacks.

Subsequently, 10 mg of teliospores were placed in a 1.5 mL centrifuge tube and mixed well with 1 mL sterile water, followed by centrifugation at 4000 rpm for 2 min at 4°C (ST16R, Thermo Fisher SCIENTIFIC, Braunschweig, Germany) and the supernatant was removed along with any floating debris. The precipitated teliospores were treated with 2% chloramine T for 15 min, followed by centrifugation as described, after which the supernatant was again removed. The teliospores were then washed three times with distilled water with centrifugation between each wash. A small portion of the teliospores was then plated on YEPS medium (2% peptone, 2% sucrose, 1% yeast extract, and 2% agar powder) amended with 30 mg penicillin and 200 mg streptomycin per liter, and the plates were cultured at 28°C under a 12 h light/dark cycle (Martinez et al., 2001). The viability of teliospores was evaluated after 3 d of incubation by monitoring the number of germinated spores out of at least 100 spores in each of four plates. Germination was visually determined using a compound microscope and teliospores were counted as germinated when the length of the germ-tube was longer than one half of the spore diameter. The viability test was repeated three times and the percentage of teliospore germination was calculated based on the collective observations.

### Evaluation of Polymerase Chain Reaction Primers for *Sporisorium reilianum* Amplification

Two sets of primers were compared to select a set of appropriate PCR primers which provided the best PCR amplification results using *S. reilianum* genomic DNA as a substrate. The sequences of the primer pair, YGSP1/GSP2, designed by Ren et al. (2008), are YGSP1 (5′-TCGCCG ACGGATGATAATCG-3′) and GSP2 (5′-GAGTCACCCGCCCAAAGTTA-3). The sequences of the SR3 primer pair, designed by Xu et al. (1999), are SR3-F (5′-GCAGCCTCAGCATTACT C3′) and SR3-R (5′-ATACACCTGTGACGGCTG-3). Genomic DNA was extracted from *S. reilianum* mycelia, cultured as previously described, using an Ezup column fungal genomic DNA extraction kit (Sangon Biotech Co., Ltd., Shanghai, China), and used as a template for PCR amplification with the designated primers. The primers were synthesized by Biotech Co. Ltd. (Shanghai, China). The reaction system and procedure are listed in Table 1. PCR amplification was conducted in a Bio-Rad C1000 Touch^TM^ Thermal Cycler (Bio-Rad, Hemel Hempstead, United Kingdom). The amplified products were assessed on a 1% agarose gel using a PowerPac electrophoresis (Bio-rad Laboratories, Inc., United States) unit.

**TABLE 1:** Reaction systems and PCR protocols using YGSP and SR3 primer pairs for the detection of *S. reilianum* in sorghum seedlings.

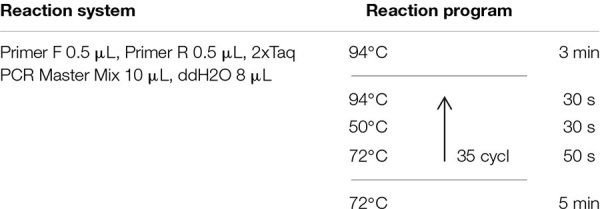

The minimal concentration of fungal DNA needed for amplification using the designed primer pairs was also assessed. The concentration of DNA in the extracted DNA solution was quantified using an ultra-low volume spectrophotometer. In brief, 1 μL of the extracted DNA solution was diluted with 9 μL sterile water, followed by six serial dilutions of 1 in 10. The diluted DNA solutions were then used as a template for PCR amplification with the designated primer pairs. The amplified products were visualized by 1% gel electrophoresis.

### Seed Treatments

Five concentrations (0.15, 0.30, 0.45, 0.60, and 0.75 μg/mL) of tebuconazole were prepared based on a preliminary assay. The level of inhibition determined in the preliminary test at the lowest concentration of tebuconazole was about 10%, while the inhibition level at the highest concentration was about 90%. A stock solution was prepared by dissolving the fungicide in acetone, along with Tween80 as an emulsifier, and then adding water. A total of 50 seeds were soaked in 10 mL of the prepared fungicide solutions for 12 h and then removed and air dried in preparation for subsequent use. Three replicate lots of seed were treated at each fungicide concentration and a solution containing acetone and Tween80, but no fungicide, was used as a control.

### Laboratory Assays of Disease Incidence

The soil used for planting the sorghum was a light sandy clay collected from the teaching farm of Guizhou University and had a pH of 5.7 (H_2_O). The soil was autoclaved for sterilization and then sifted for use.

The main factors affecting S. reilianum infection are soil moisture and temperature (Baier and Kruger, 1962; Matyac and Kommedahl, 1985b). Therefore, the incubation conditions were optimized by adjusting the soil moisture, temperature, and spore quantity present in the soil. The optimized protocol for obtaining the highest level of disease incidence was as follows: Soil was mixed with 0.2% (by weight) teliospores and then added to a plug-designed planting tray. Sterilized sorghum seeds were then planted at 2 seeds per well. The seeds were placed 2 cm deep in the pathogen-contaminated soil. The plug trays were then placed in an incubator and cultured at 10°C, 60% humidity, and 20% soil moisture. The trays were then transferred to a greenhouse set at 25°C and a 12 h light/dark cycle. Seedlings were collected after seven days when they were 2–3 cm in length. A total of 18 similar-sized seedlings were collected from each replicate tray and used for the extraction of genomic DNA.

### Extraction of Genomic DNA From the Sorghum Seedlings

Eighteen seedlings were used within each replicate for DNA extraction. Genomic DNA was extracted from individual seedlings using a DNA extraction kit (Sangon Biotech Co., Ltd., Shanghai, China). Seedling were cleaned of visible soil by rinsing under running tap water and then surface disinfected by submersion in 10% NaOCl solution with agitation for 2 min. Plants were rinsed twice in sterile water for 30 s each time (Anderson et al., 2015). Seedling were placed in a sterilized mortar, ground into a powder with a pestle in liquid nitrogen, and then immediately transferred into a 1.5 mL Eppendorf tube. Next, 800 μL of CTAB extraction buffer solution (preheated in a water bath to 65°C) was added to each of the tubes containing the ground seedling tissues and the mixture was then thoroughly mixed several times on a vortexer (5 min/time) for a total of 20 min. The seedlings were subsequently centrifuged for 2 min (12000 rpm, 4°C). The supernatant was then removed and mixed with an equal volume of phenol: chloroform (1:1, 400 μL each) and the mixture was then centrifuged for 10 min. The resulting supernatant was then mixed with an equal volume of chloroform, followed by centrifugation for 2 min. This process was repeated several times until no protein layer was visible. Genomic DNA was precipitated from supernatant by addition of 2 volumes of ice-cold absolute ethanol and storage at −20°C for 1 h, followed by centrifugation for 2 min. The resulting supernatant was discarded, and the precipitated samples of genomic DNA were washed with 70% ethanol, precipitated twice, dried at room temperature, and finally dissolved in 50 μL of DEPC treated water for subsequent use.

### PCR Detection

The genomic DNA obtained from sorghum seedlings was used as a template for PCR amplification using the SR3 primers. The reaction system and procedure are provided in Table 1. The PCR products were detected using 1% agarose gel electrophoresis. The incidence of infection of seedlings by the smut pathogen S. reilianum was determined by the analysis of bands present on the agarose gels.

### Evaluation of Fungicide Efficacy by Inhibition of Teliospore Germination

Inhibition of spore germination is a common method to determine the efficacy of fungicides. Therefore, the EC_50_ and EC_95_ of tebuconazole were determined by measuring teliospore germination after exposure to the prepared solutions of tebuconazole.

The teliospores were fumigated for 45 min with a mixture of 10 mL/m^3^ 40% formaldehyde and 5 g/m^3^ potassium permanganate (Liu et al., 2007). Spore suspensions were prepared with sterile water and adjusted to about 100 spores per field of microscope. A tebuconazole acetone solution was added to sterilized and slightly cooled YEPS medium (pH = 7.0) and fully mixed. Medium amended with an equal amount of acetone was used as a control. Three replicates were used for each concentration of fungicide. Five concentrations of fungicide (0.02, 0.04, 0.08, 0.16, and 0.32 μg/mL) were used based on a preliminary assay. The level of inhibition determined in the preliminary test at the lowest concentration of tebuconazole was about 10%, while the inhibition level at the highest concentration was about 90%. A 0.1 mL drop of spore suspension was evenly distributed on the surface of each YEPS plate (9 cm), and the plates were then placed in an incubator at 28°C (Jiang et al., 2004). The rate of spore germination was determined after 48 h of culture by counting 100 spores in three different fields of view (300 spores per plate). A spore was considered germinated when the length of the germ-tube was longer than one half of the spore diameter.

### Data Analysis

The level of infection and teliospore germination were calculated using the following equation:


$$\mathrm{Inhibition}\,\mathrm{rate}=\frac{\text{C}\,\text{K}-\text{T}}{\text{C}\,\text{K}}\times 100\%$$

T and CK infection in the seedling assay were determined by a positive PCR amplification in the treated and control seedlings. T and CK in the teliospore germination assay were determined by observations of spore germination in the treated and control teliospores.

The EC_50_ and EC_95_ values were calculated by regressing the percentage inhibition (probability value transformed) against the logarithm value of the fungicide concentration (log transformed). Regression analysis was performed using Microsoft Excel 2007 software (Li et al., 2015).

### Analysis of Variance

Analysis of Variance between treatments was analyzed using the DPS^®^ (Data Processing System, version7.05; Hangzhou RuiFeng Information Technology Co. Ltd., Hangzhou, China) software.

## Results

### *Sporisorium reilianum* Colony Morphology

**FIGURE 2:**
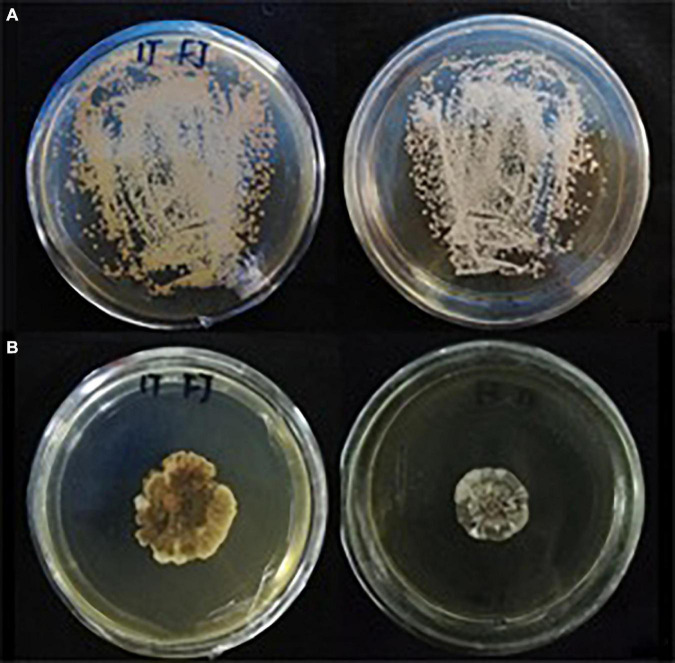
Morphology of S. reilianum colonies growing on YEPS medium at 28°C with a 12 h light/dark cycle. **(A)** a small amount of teliospore suspension was distributed on YEPS medium and cultured for 10 d; **(B)** single colony cultured on YEPS medium for 30 d.

Many small colonies of *S. reilianum* were present on the YEPS medium after incubation for 10 days (Figure 2A). A single colony was selected and cultured on YEPS medium to make observations of colony morphology (Figure 2B). All the morphological observations of colony appearance were consistent with the description of *S. reilianum* in the *Manual of Fungal Identification* (Wei, 1979). The percentage of teliospore germination was 67.5%.

### PCR-Amplification of *Sporisorium reilianum* Genomic DNA With Select Primers

**FIGURE 3:**
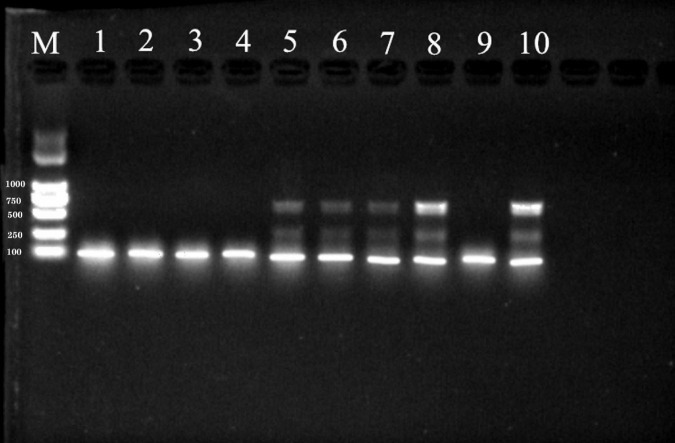
PCR products amplified from genomic DNA of *S. reilianum* using YGSP (lane 1–5) and SR3 (lane 6–10) primer pairs, subsequently separated on a 1% agarose gel. Lane M is DNA ladder DL2000.

The expected band size by SR3 primer was 680 bp. The PCR products amplified by SR3 yielded four clear, distinct bands on an agarose gel (Figure 3). In contrast, only one product was generated by the PCR-amplification using YGSP primers, evidenced as a single band with low definition on an agarose gel. As a result, the SR3 primers were selected for assessment of the presence of *S. reilianum*, based on the clarity of the bands that were produced by PCR-amplification of *S. reilianum* genomic DNA.

### Limit of Detection of *Sporisorium reilianum* Genomic DNA by PCR

**FIGURE 4:**
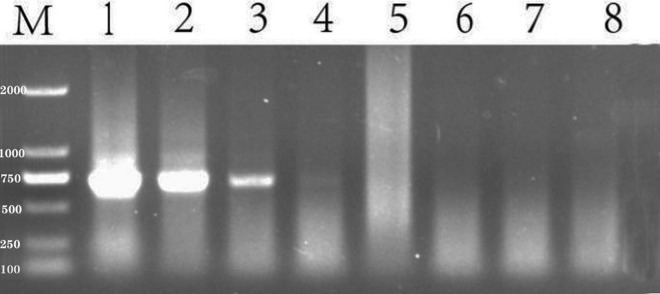
Sensitivity of detection of *S. reilianum* using the SR3 primer pair. PCR products amplified from a 10-fold dilution series of *S. reilianum* genomic DNA and visualized on a 1% agarose gel. Lane 1:107 ng/μL, Lane 2: 10.7 ng/μL, Lane 3: 1.07 ng/μL, Lane 4: 1.07 × 10^–1^ ng/μL, Lane 5: 1.07 × 10^–2^ ng/μL, Lane 6:1.07 × 10^–3^ ng/μL, Lane 7:1.07 × 10^–4^ ng/μL. Lane M: DNA ladder DL2000.

The concentration of the extracted, genomic DNA of *S. reilianum* was 107 ng/μL, which was diluted in 10-fold dilution series to obtain concentrations of 10.7 ng/μL, 1.07 ng/μL, 1.07 × 10^–1^ ng/μL, 1.07 × 10^–2^ ng/μL, 1.07 × 10^–3^ ng/μL, and 1.07 × 10^–4^ ng/μL. The various concentrations *S. reilianum* genomic DNA were then used as a template for PCR-amplification with the SR3 primers. As shown in Figure 4, the designated target band was amplified and visible on an agarose gel at concentrations of 10.7 ng/μL, 1.07 ng/μL, and 1.07 × 10^–1^ ng/μL of *S. reilianum* genomic DNA. The band size was approximately 680 bp. The amplification using the 10.7 ng/μL concentration of genomic DNA produced the strongest (brightest) band, while concentrations of 1.07 × 10^–2^ ng/μL, 1.07 × 10^–3^ ng/μL, and 1.07 × 10^–4^ ng/μL did not produce any visible PCR products. Therefore, a concentration of 1.07 ng/μL genomic DNA was considered to be the limit for the detection of *S. reilianum.*

### Efficacy of Seed Treatments Against *Sporisorium reilianum* Infection Determined by PCR

**FIGURE 5:**
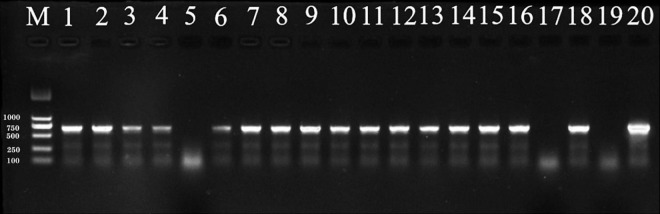
PCR products amplified from DNA extracted from sorghum seedings and visualized by 1% agarose gel electrophoresis. Sorghum seeds were planted in soil containing 0.2% (by weight) teliospores of *S. reilianum*. After 3 days of incubation at 10°C, 20% soil moisture, and 60% relative humidity, the planted seed trays were transferred to a greenhouse (25°C and a 12-h light/dark cycle) for 4 days after which the seedlings were collected for DNA extraction. Lane M: DNA ladder DL2000. Lane 1–18: sorghum seedings. 19: negative control (Seedlings from pathogen-free soil); 20: positive control (DNA from *S. reilianum* mycelia).

The incidence of pathogen infection of sorghum seedlings was assessed after seven days of seedling growth. Incidence was determined to be positive if *S. reilianum* was detected by PCR, while negative PCR results were considered to be a non-infected seedling (Figure 5). Treatment efficacy was determined by the number of negative vs. positive PCR results. The fewer positive PCRs observed, the better the efficacy of the fungicidal treatment.

**FIGURE 6:**
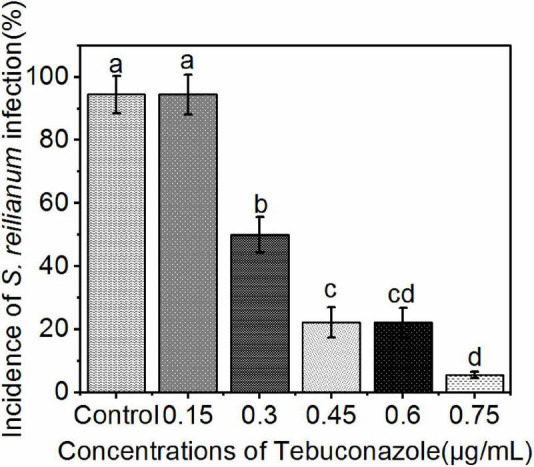
Incidence of *S. reilianum* infection in sorghum seedlings determined by positive PCR reactions. Sorghum seeds were treated with five different concentrations of tebuconazole (x-axis), then planted in soil containing 0.2% (by weight) teliospores of *S. reilianum* and grown 7 days. Each bar represents the mean ± se (*n* = 3) level of disease incidence (y-axis). Different lowercase letters indicate a significant difference between the different concentration of tebuconazole (*p* < 0.05).

The incidence of infection in seeds treated with 0.15, 0.30, 0.45, 0.60, and 0.75 μg/mL tebuconazole was 83.33%, 55.56%, 27.78%, 16.67%, respectively, while the incidence of infection in the untreated control was 88.88% (Figure 6). Conversely, the infection inhibition rate was 6.24%, 37.48%, 67.74%, 81.24, and 93.74%, respectively.

A linear regression of the level of inhibition vs. the logarithmic concentration of tebuconazole, yielded the equation y = 6.9494+4.2741x (*r* = 0.9953). Based on this equation, the EC_50_ value was calculated to be 0.35 μg/mL tebuconazole (95% confidence limit: 0.33–0.37 μg/mL), and the EC_95_ was 0.85 μg/mL (95% confidence limit: 0.78–0.92 μg/mL).

### Efficacy of Tebuconazole Against *Sporisorium reilianum* Determined by Inhibition of Spore Germination

**FIGURE 7:**
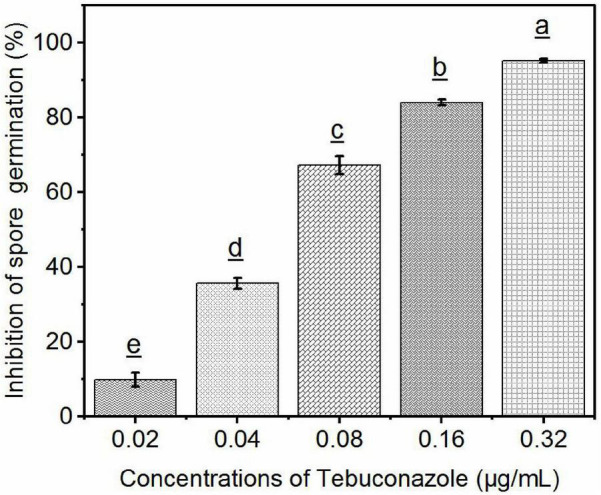
Efficacy of tebuconazole against *S. reilianum* determined by inhibition of spore germination. Spore suspension was evenly distributed on the surface of YEPS plate containing different concentrations of fungicides, after 48 h of culture at 28°C, the rate of spore germination was determined by counting 100 spores in three different fields of view (300 spores per plate). A spore was considered germinated when the length of the germ-tube was longer than one half of the spore diameter. Each bar represents the mean ± se (*n* = 3). Different lowercase letters indicate a significant difference between the different concentration of tebuconazole (*p* < 0.05).

The percentage of teliospore germination after 48 h of incubation on media amended with 0.02, 0.04, 0.08, 0.16, and 0.32 μg/mL tebuconazole was 73.58%, 52.44%, 26.83%, 13.00%, and 4.06%, respectively. The germination on media without tebuconazole was 82.00%. Conversely, the teliospore germination inhibition rate was 10.27%, 47.56%, 67.28%, 84.14, and 95.04%, respectively (Figure 7).

Linear regression of the level of inhibition vs. the logarithmic concentration of tebuconazole yielded the equation y = 7.8689+2.2968x (*r* = 0.9837). Based on this equation, the EC_50_ value was calculated to be 0.056 μg/mL (95% confidence limit: 0.046–0. 070 μg/mL) and the EC_95_ was 0.293 μg/mL (95% confidence limit: 0.210–0.410 μg/mL).

## Discussion

The efficacy of fungicides can be tested under either laboratory or field conditions. Inhibition of spore germination and mycelial growth are typically the parameters measured under laboratory conditions while a disease index is calculated to determine fungicidal efficacy in the field.

Tebuconazole is one of the most commonly used fungicides in agriculture and efficacy under laboratory conditions has been extensively examined. For example, the EC_50_ and EC_90_ values, based on the inhibition of mycelial growth, for *Alternaria mali* are 0.034 and 0.587 μg/mL, and 0.019 and 0.394 μg/mL for *Physalospora. piricola*, respectively (Jian-lu et al., 2007). The mean EC_50_ of tebuconazole, based on the inhibition of mycelial growth of 10 *Fusarium graminearum* isolates, was reported to be 0.19 mg/L (Avozani et al., 2014). The EC_50_ and EC_90_ values for 43% tebuconazole suspension concentrates against *Phomopsis aspara*g*i* is 7.4241 and 47.1322 mg/L, respectively (Jia et al., 2009). EC_50_ and EC_95_ of tebuconazole for sensitive strains of *Sclerotinia homoeocarpa* is 0.078 and 1.943 μg/mL, respectively (Couch, 2003).

In our study, a PCR method was used to determine infection incidence of *S. reilianum* in sorghum seedlings. Based on our dose–response data, we performed a linear regression and determined an EC_50_ (0.35 μg/mL) and an EC_95_ (0.85 μg/mL) value for tebuconazole using the derived regression equation. The EC_50_ value for tebuconazole determined by PCR was higher than most it was in reports of several other pathogens that determined efficacy based on the inhibition of mycelial growth and/or teliospore germination. Our present results suggest that inhibition of spore germination or mycelial growth alone may not completely reflect efficacy. Spores in the soil that are not in direct contact with the seed may still germinate and their mycelia may infect the germinated seedling, even though mycelial growth may be inhibited. Thus, in actuality a higher concentration is needed to prevent infection than may be indicated in a petri dish assay. EC_50_ (0.056 μg/mL) and EC_95_ (0.293 μg/mL) value determined by inhibition of spore germination were all lower than these determined by PCR method. This result was consistent with our conclusion above. Therefore, we assume that our calculations are reliable under the conditions used in our study.

An important objective in our study was to improve the level of infection achieved in untreated, control seedlings. Methods for artificial inoculation using *S. reilianum* have been previously reported (Xiaowei Zhou et al., 2017). Four methods were used to render different maize varieties highly susceptible to infection by *S. reilianum*. The methods included injection of coleoptiles, mixing teliospores in the soil, root irrigation with water containing teliospores, and soaking seeds in water containing teliospores. Their results revealed that injection of corn coleoptiles provided the highest levels of infection. Since the purpose of our study was to evaluate the efficacy of tebuconazole as a seed treatment, the method of mixing the soil with teliospores was utilized, as it also more closely reflects field conditions.

The optimal temperature for infection of sorghum by *S. reilianum* has been reported to be 21∼28°C, with a low to medium level of soil moisture (Wu et al., 1981). It has reported that *S. reilianum* infection was the highest when soil moisture content was 15.5%. In the present study, a 0.2% concentration of teliospores in soil and a 20% of soil moisture level provided a high rate of infection. Notably, a previous study indicated that a suitable soil temperature for *S. reilianum* infection was 20∼30°C, with 35°C as the highest temperature and 10°C as the lowest temperature (Zhang, 1980; Wang et al., 2002). The approach used in the present study to achieve a high rate of infection was as follows. Seedlings were first placed at the minimum temperature of 10°C for 3 days and then transferred to 25°C. The objective of placing the seedlings at 10°C for the first three days was to stress the plants and make them susceptible to pathogen infection. The rate of infection achieved in our study was 88.88%, which is consistent with the results reported by Craig and Frederiksen (1992) and Jiang et al. (2014).

Modern approaches for detecting fungal plant pathogens utilize high-throughput molecular strategies. These include qualitative polymerase chain reaction (qPCR), real-time, quantitative PCR (RT-qPCR), nested PCR, loop-mediated isothermal amplification (LAMP), rolling circle amplification (RCA), and nucleic acid sequence-based amplification (NASBA) (Hariharan and Prasannath, 2021). Thus, PCR has become an essential diagnostic tool.

Real-time, quantitative PCR (RT-qPCR) has become increasingly used in plant pathogen diagnostics (Mirmajlessi et al., 2015). An advantage of RT-qPCR over qPCR is that the former is quantitative and can provide an estimate of the level of infection (i.e., how much pathogen is present) (McCartney et al., 2003). In our study, however, qPCR was selected as a diagnostic tool since we were simply trying to determine infection incidence, so a quantitative assessment was not required, and because qPCR is easier to conduct than RT-qPCR, the latter of which also requires greater expertise. In summary, due to the slow growth of mycelia and the low germination rate of teliospores of *S. reilianum*, as well as the time-consuming nature of field observations and evaluations, PCR-based evaluation of sorghum seedlings provides an excellent approach for evaluating the efficacy of fungicide-based seed treatments in preventing *S. reilianum* infection of sorghum seedlings.

## Data Availability Statement

The original contributions presented in the study are included in the article/supplementary material, further inquiries can be directed to the corresponding authors.

## Author Contributions

HQ and AH contributed to conception and design of the study. ZZ performed the experiments, analysis, and wrote first draft of the manuscript. JF and MF validated the methodology, analysis, and provided supervision. All authors contributed to manuscript revision and read and approved the submitted version.

## Conflict of Interest

The authors declare that the research was conducted in the absence of any commercial or financial relationships that could be construed as a potential conflict of interest.

## Publisher’s Note

All claims expressed in this article are solely those of the authors and do not necessarily represent those of their affiliated organizations, or those of the publisher, the editors and the reviewers. Any product that may be evaluated in this article, or claim that may be made by its manufacturer, is not guaranteed or endorsed by the publisher.
